# Association of a single nucleotide polymorphism in *titin *gene with marbling in Japanese Black beef cattle

**DOI:** 10.1186/1756-0500-2-78

**Published:** 2009-05-07

**Authors:** Takahisa Yamada, Seiki Sasaki, Shin Sukegawa, Sachiyo Yoshioka, Youichi Takahagi, Mitsuo Morita, Hiroshi Murakami, Fumiki Morimatsu, Tatsuo Fujita, Takeshi Miyake, Yoshiyuki Sasaki

**Affiliations:** 1 Laboratory of Animal Breeding and Genetics, Graduate School of Agriculture, Kyoto University, Sakyo-ku, Kyoto 606-8502, Japan; 2 Maebashi Institute of Animal Science, Livestock Improvement Association of Japan, Maebashi, Gunma 371-0121, Japan; 3 Research and Development Center, Nippon Meat Packers, Inc., Tsukuba, Ibaraki 300-2646, Japan; 4 Kyoto Cattle Genetics Section, Beef Information & Genetics Institute, Inc, Otsu, Shiga 520-0865, Japan; 5 Cattle Embryo Transfer & Genetics Section, Oita Prefectural Institute of Animal Industry, Takeda, Oita 878-0201, Japan

## Abstract

**Background:**

Marbling defined by the amount and distribution of intramuscular fat is an economically important trait of beef cattle in Japan. We have recently reported that single nucleotide polymorphisms (SNPs) in the *endothelial differentiation*, *sphingolipid G-protein-coupled receptor*, *1 *(*EDG1*) gene were associated with marbling in Japanese Black beef cattle. As well as *EDG1*, the *titin *(*TTN*) gene, involved in myofibrillogenesis, has been previously shown to possess expression difference in *musculus longissimus *muscle between low-marbled and high-marbled steer groups, and to be located within genomic region of a quantitative trait locus for marbling. Thus *TTN *was considered as a positional functional candidate for the gene responsible for marbling. In this study, we explored SNP in *TTN *and analyzed association of the SNP with marbling.

**Findings:**

A SNP in the promoter region of *TTN*, referred to as *g.231054C>T*, was the only difference detected between high- and low-marbled steer groups. The SNP was associated with marbling in 3 experiments using 101 sires (*P *= 0.004), 848 paternal half-sib progeny steers from 5 sires heterozygous for the *g.231054C>T *(*P *= 0.046), and 820 paternal half-sib progeny steers from 3 sires homozygous for *C *allele at the *g.231054C>T *(*P *= 0.051), in Japanese Black beef cattle. The effect of genotypes of the SNP on subcutaneous fat thickness was not statistically significant (*P *> 0.05).

**Conclusion:**

These findings suggest that in addition to the *EDG1 *SNPs, the *TTN *SNP polymorphism is associated with marbling and may be useful for effective marker-assisted selection to increase the levels of marbling in Japanese Black beef cattle. Further replicate studies will be needed to confirm the allelic association observed here, and to expand the results to evaluate all possible genotypic combinations of alleles.

## Background

Marbling has an important effect on the economics of beef production [1-4]. Thus, it is greatly interesting to obtain better knowledge on the molecular architecture of marbling and to generate new opportunities for more effective marker-assisted selection.

The *endothelial differentiation*, *sphingolipid G-protein-coupled receptor*, *1 *(*EDG1*) gene, which is involved in blood vessel formation [5], exhibited higher expression levels in high-marbled steer group than in low-marbled steer group in *musculus longissimus *muscle across all ages of the test period [6,7]. We have recently showed that single nucleotide polymorphisms (SNPs) in the *EDG1 *were associated with marbling in Japanese Black beef cattle [8].

From the result of our previous differential-display PCR (ddPCR) analysis in *musculus longissimus *muscle [6], the *titin *(*TTN*) gene exhibited higher expression levels in low-marbled steer group than in high-marbled steer group in the late stage of the test period. Since *TTN *is known to be involved in myofibrillogenesis [9], the differential expression suggested that the decrease in the *TTN *expression might promote proliferation, differentiation, or maturation of adipocyte-lineage cells by weakening the structural integrity of the sarcomere, thereby resulting in high levels of marbling.

We have also located the *TTN *gene within genomic region of a quantitative trait locus for marbling [10,11], which is mapped in a half-sib family of Japanese Black cattle to bovine chromosome 2 region [12]. Thus, the *TTN *gene was regarded as a positional functional candidate for the gene responsible for marbling.

### *TTN *gene structure

We screened the NCBI bovine genome sequence database (National Center for Biotechnology Information, Bethesda, MD) with the publicly available bovine *TTN *cDNA sequence [GenBank:XR_027685] including 101,593 bp, and obtained 5,046,004-bp bovine genomic sequence [GenBank:NW_001494580] containing the *TTN *gene. By comparing the genomic and cDNA sequences, we showed that the bovine *TTN *gene spans 276,648 bp and comprises 211 bp of the first exon, 1,315 bp of the last exon, 100,067 bp of 313 internal exons, and 175,055 bp of 314 introns.

### SNP detection

Two Holstein steers and two somatic nuclear-derived cloned steers [13] from a Japanese Black Itofuku sire with a very high estimated breeding value for marbling [14], which were assigned for low-marbled and high-marbled steer groups, respectively, in our previous ddPCR analysis [6], were used for SNP detection in this study. The two low-marbled Holstein steers and two high-marbled cloned steers were previously shown to have different *TTN *gene expression patterns [6]. The details of DNA samples from these steers were described previously [6]. We used two high-marbled cloned steers to confirm the authenticity of newly discovered SNP in the *TTN *gene.

PCR primers were designed to target ~7-kb proximal promoter and the first and last exon regions, to screen polymorphisms in the *TTN *gene between two low-marbled Holstein steers and two high-marbled cloned steers (Table 1). We selected these genomic regions for SNP detection, because it is likely that these regions are involved in *TTN *gene expression. PCR amplifications were performed using 25 ng of the prepared DNA as template in a final volume of 100 μl containing 1 μM of each primer, 0.25 mM of each dNTP, 2.5 U of Go *Taq *polymerase (Promega, Madison, WI), and 1 × Go *Taq *buffer (Promega). The PCR conditions were carried out as follows: 94°C for 2 min, 35 cycles of 94°C for 30 s, the annealing temperature indicated in Table 1 for 30 s, and 72°C for 1 min, followed by a further 5-min extension at 72°C. PCR products were examined by electrophoresis through a 1.0% agarose gel to determine the quality and quantity for DNA sequencing. DNA sequencing of PCR-amplified products was performed by the direct sequencing with an ABI377 sequencer (ABI, Foster City, CA) following standard Big Dye protocols (ABI). Primers used for PCR amplifications and obtained from primer walking were used as sequencing primers. Nucleotide polymorphisms were identified by comparison of sequence traces between the 2 steer groups, and designated according to nomenclature for the description of sequence variations in the HGVS (Human Genome Variation Society, Fitzroy, VIC, Australia) [15].

**Table 1:** Primers designed for SNP detection in the bovine *TTN *gene

Primer name	Sequence	Nucleotide position*	Annealing temperature
TT-1F	5'-TGCAGCCAAAGATGTAGAAGC-3'	-7,203 to -7,183	60°C
TT-1R	5'-TCAGTCCTTCCAATGAATACCC-3'	-5,262 to -5,283	60°C
TT-2F	5'-GTCATGTATGGATGAGAGTTGGA-3'	-5,428 to -5,406	59°C
TT-2R	5'-GAGAGTCGGACACGACTGAA-3'	-3,487 to -3,506	59°C
TT-3F	5'-GCCATTAAATGTGTGCGTGA-3'	-3,618 to -3,599	60°C
TT-3R	5'-TTGTGCACGTGTGTGTATGG-3'	-1,674 to -1,693	60°C
TT-4F	5'-TTCCCATAAGAATTAAAGTGGTTTG-3'	-1,806 to -1,782	59°C
TT-4R	5'-GATAGCACCAAGTAGTCCGACA-3'	329 to 308	59°C
TT-5F	5'-CCTAAGAGGTGAGCAATCCA-3'	275,190 to 275,209	58°C
TT-5R	5'-GCTGTAGAGAACTTGAGTTGC-3'	276,756 to 276,736	58°C

*The nucleotide positions of primers are shown according to their location relative to the putative transcription initiation site of the *TTN *gene.

We sequenced the 6,615-bp 5' flanking region, the 211-bp first exon region, and the 1,315-bp last exon region of the *TTN *gene for the 4 DNA samples. This sequence analysis revealed only one SNP in the *TTN *gene between the 2 steer groups: a C to T transition located 652 bp upstream of the putative transcription initiation site in the promoter region (*g.231054C>T*). The *g.231054C>T *has been submitted to the dbSNP with ss accession number 120037472. The two Holstein steers were homozygous for *C *allele at the *g.231054C>T *site, whereas the two cloned steers heterozygous for *C *and *T *alleles at the *g.231054C>T *site.

### Association study

#### Samples and data

We performed 3 experiments for association of the *g.231054C>T *with marbling and subcutaneous fat thickness. We used 101 Japanese Black sires in experiment 1. The sires used to be or are used in the Oita Prefectural Institute of Animal Industry (Oita, Japan). There was no strong bias for a specific father or a specific maternal grandfather of the sires, and the sire panel likely represents a variety of the sire lines. In experiment 2, 848 paternal half-sib Japanese Black progeny steers (55 to 445 steers per sire) produced from 5 sires heterozygous for the *g.231054C>T*, with dams considered to represent a random sample of the female population, were used. We used 820 paternal half-sib Japanese Black progeny steers (88 to 535 steers per sire) produced from 3 sires homozygous for *C *allele at the *g.231054C>T*, with dams considered to be a random mating population, in experiment 3. These progeny steers (in experiments 2 and 3) were fattened, and shipped to the carcass market in the Oita prefecture. Semen or blood of the sires and adipose tissues of the progeny steers were collected for SNP genotyping. DNA samples were prepared from the materials according to standard protocols.

The predicted breeding values of the sires and the progeny steers for marbling score and subcutaneous fat thickness were obtained from the Oita recording system for beef cattle reported by Sasaki *et al*. [16], and used as phenotypic values in this study. In the recording system, the breeding values were predicted from carcass records of Japanese Black steers and heifers, fattened in the Oita prefecture. The fattened animals were shipped to various carcass markets from 1988 to 2003, where they were slaughtered and their carcasses evaluated. The data were edited to connect across subclasses such that each market-year subclass had 50 or more animals and each farm had 10 or more animals. The final number of animals was 48,045, and there were 89 market-year subclasses, 332 farms, and 228 sires.

Marbling score and subcutaneous fat thickness were measured on carcasses dissected at the sixth and seventh rib section, according to the Japanese meat grading system by certified graders from the Japan Meat Grading Association (Tokyo, Japan) [4]. Marbling score was scored from 1 to 12 (beef marbling standard number) with a standard model panel, in which higher scores correspond to more marbling. Score 1 likely corresponds to "Practically Devoid", "Traces", "Slight" and "Small", score 2 or 3 "Modest", "Moderate" and "Slightly Abundant", and score 4 or 5 "Moderately Abundant". Subcutaneous fat thickness was measured at the rib interface perpendicular to the outside surface at a point 3-fourths the length of the *longissimus *muscle from its chine bone end.

Data were analyzed by the REML method using the MTDFREML programs [17], and genetic and environmental variances were estimated. The BLUP option in the programs using the estimated variance components was chosen to predict the breeding values of animals with a single trait model. Sex, market-year, and farm were considered fixed effects. Fattening period and slaughter age were also considered as up to quadratic covariates. The fattening period denotes the period from the start of fattening to shipping to market for each animal. These fixed effects were all significant (*P *< 0.001). Random effects included additive genetic effect of the individuals; that is, the animal model was adopted to predict the breeding values.

This study conformed to the guidelines for animal experimentation of the Graduate School of Agriculture, Kyoto University (Kyoto, Japan).

#### SNP genotyping

The *g.231054C>T *was genotyped using PCR-restriction fragment length polymorphism (RFLP) method. PCR primers used for PCR-RFLP were 5'-TCATCTCCTAACTACTTCCCA-3' and 5'-ACAAAATCTGAACCTGGCTT-3' (Nucleotide positions relative to the putative transcription initiation site of the *TTN *gene were -842 to -822 and -616 to -635, respectively). PCR amplifications were carried out as described in SNP detection section, using a final volume of 20 μl and the annealing temperature of 55°C. An aliquot of PCR-amplified products was digested at 37°C for 1 h with restriction enzyme *Hpy*CH4III, and electrophoresed on a 3.0% agarose gel. Agarose gels were stained with ethidium bromide and photographed under an ultraviolet light. Using this method, 227-bp PCR fragments containing the SNP site were amplified, and *Hpy*CH4III-digested into 36- and 191-bp fragments at the *C *allele, but not at the *T *allele: the *CC *homozygotes, the *TT *homozygotes, and the *CT *heterozygotes resulted in 2 bands (36 and 191 bp), 1 band (227 bp), and 3 bands (36, 191, and 227 bp).

#### Experiment 1

Genotyping 101 sires for the *g.231054C>T *revealed 50 animals homozygous for *C *allele, 47 animals heterozygous for *C *allele and *T *allele, and 4 animals homozygous for *T *allele. The 4 *TT *homozygotes were too small sample size to give reliable estimates, and then excluded from our statistical analysis. Statistically significant differences among the genotypes of the SNP were detected for marbling (*P *= 0.004), but not for subcutaneous fat thickness (*P *= 0.984), by the analysis with the model that included the SNP genotype as the fixed effect and the sire (father of the sire) as the random effect (Table 2). The marbling score was significantly higher in the *CT *heterozygotes than in the *CC *homozygotes (Table 2).

**Table 2:** Effect of the SNP genotypes on marbling and subcutaneous fat thickness in experiment 1

		Breeding value*	
		
Genotype	No. of animals	Marbling score	Subcutaneous fat thickness (mm)
*CT*	47	2.81^a ^± 0.25	-2.85 ± 0.74
*CC*	50	2.13^b ^± 0.23	-2.84 ± 0.80

*The breeding values are given as estimates ± SE.

^a, b^Estimates at different genotypes without a common letter in their superscripts significantly differ (*P *< 0.01).

#### Experiment 2

To better estimate the effect of the *g.231054C>T T *allele on marbling and subcutaneous fat thickness, we performed experiment 2. The interaction between the SNP genotype and the sire was not statistically significant (*P *= 0.320 for marbling, and *P *= 0.192 for subcutaneous fat thickness) in the model that included the SNP genotype and the sire as the fixed effects and their interaction, and was excluded from our statistical model. In the model without the interaction effect, the SNP genotype had the statistically significant effect on marbling (*P *= 0.046), but not subcutaneous fat thickness (*P *= 0.749) (Table 3). The marbling score was significantly higher in the *TT *homozygotes than in the *CC *homozygotes, and that of the heterozygotes intermediate between those of the 2 homozygotes (Table 3).

**Table 3:** Effect of the SNP genotypes on marbling and subcutaneous fat thickness in experiment 2

		Breeding value*	
		
Genotype	No. of animals	Marbling score	Subcutaneous fat thickness (mm)
*TT*	149	3.42^a ^± 0.08	-0.33 ± 0.36
*CT*	378	3.22^b ^± 0.06	-0.22 ± 0.25
*CC*	321	3.19^b ^± 0.06	-0.05 ± 0.27

*The breeding values are given as least squares means ± SE.

^a, b^Means at different genotypes without a common letter in their superscripts significantly differ (*P *< 0.05).

#### Experiment 3

To further verify the association of the *g.231054C>T *with marbling, we carried out experiment 3. The steers in this experiment could be grouped only according to the alleles that they received from their dams, which are considered to be a random sample of a general population in Japanese Black beef cattle. Therefore, this experiment likely allowed a linkage disequilibrium estimate of the effect of the SNP. The interaction between the SNP genotype and the sire was not statistically significant (*P *= 0.208 for marbling, and *P *= 0.980 for subcutaneous fat thickness) in the model that included the SNP genotype and the sire as the fixed effects and their interaction, and was excluded from our statistical model. In the model without the interaction effect, the SNP genotype effect reached marginal significance (*P *= 0.051) for marbling, but not for subcutaneous fat thickness (*P *= 0.310) (Table 4). The marbling score exhibited a tendency to be higher in the *CT *heterozygotes than in the *CC *homozygotes (Table 4).

**Table 4:** Effect of the SNP genotypes on marbling and subcutaneous fat thickness in experiment 3

		Breeding value*	
		
Genotype	No. of animals	Marbling score	Subcutaneous fat thickness (mm)
*CT*	270	2.88 ± 0.06	-0.04 ± 0.24
*CC*	550	2.76 ± 0.04	0.23 ± 0.18

*The breeding values are given as least squares means ± SE.

Based on 3 experiments, we showed that the *g.231054C>T *is associated with marbling in Japanese Black beef cattle, with the *T *allele resulting in high levels of marbling. Especially, in experiments 2 and 3, because the dams can be considered to represent a random sample of the Japanese Black population, the association is likely to be true. We used the predicted breeding values as phenotypic values, and then sire contributions to the predicted breeding values were the same for all offspring, even if the heterozygous sires were used in experiment 2. Thus, it is likely to be desirable to use larger numbers of dams in the analyses using paternal half-sib progeny steers such as experiments 2 and 3. We should note that the experiments in this study were the case. Further, the association of the *g.231054C>T *with marbling was corroborated by an independent study using 104 paternal half-sib Japanese Black families with a total of 821 progeny steers (S. Sukegawa, unpublished data).

Based on the association of the *g.231054C>T *with marbling, together with *TTN *expression difference between low-marbled (with *g.231054C>T C *allele) and high-marbled steer groups (with *g.231054C>T C *and *T *alleles) and co-localization of the marbling quantitative trait locus with the *TTN*, we can hypothesize that the SNP in the promoter region might have an impact on *TTN *expression and also marbling by affecting *TTN *promoter activity. However, the SNP is not located within any of as yet identified canonical sequences involved in gene transcription. Thus, a more likely event is that the *TTN *SNP is in linkage disequilibrium with an unidentified and truly relevant mutation, rather than functional and a causal mutation for marbling.

The effect of genotypes of the SNP was not statistically significant (*P *> 0.05) for subcutaneous fat thickness. Further, the marbling quantitative trait locus corresponding to chromosomal position of the *TTN *did not have statistically significant effect on subcutaneous fat thickness [10,11]. Thus, it is likely that the *TTN *SNP is not associated with subcutaneous fat thickness in Japanese Black beef cattle. This might be supported by the fact that Japanese Black breed exhibits low genetic correlation between marbling and subcutaneous fat thickness [7].

Several previous studies have reported polymorphisms associated with beef marbling using beef cattle breeds other than Japanese Black [18-27]. We have recently reported that SNPs in the *EDG1 *gene were associated with marbling in Japanese Black breed [8]. Thus, our present study seems to be the second report to show polymorphisms associated with marbling using Japanese Black breed.

The information on the *TTN *SNP obtained in this study, as well as the *EDG1 *SNPs, may be applied to effective marker-assisted selection to increase the levels of marbling in Japanese Black beef cattle. Further replicate studies will be needed to confirm the allelic association observed here, and to expand the results to evaluate all possible genotypic combinations of alleles.

## Competing interests

The authors declare that they have no competing interests.

## Authors' contributions

TY carried out the statistical analyses and drafted the manuscript. SS, SS, SY, and YT carried out the SNP detection and genotyping. MM, HM, FM, and TF participated in the sample and data collection. TM carried out further statistical analyses. YS participated in the design and coordination of the study and helped to draft the manuscript. All authors read and approved the final manuscript.
